# *Ganoderma lucidum*, a promising agent possessing antioxidant and anti-inflammatory effects for treating calvarial defects with graft application in rats^1^

**DOI:** 10.1590/s0102-865020190090000004

**Published:** 2019-11-25

**Authors:** Nihat Laçin, Serhat Bozan İzol, Fikret İpek, Mehmet Cudi Tuncer

**Affiliations:** I PhD, Assistant Professor, Department of Oral and Maxillofacial Surgery, Faculty of Dentistry, University of Katip Çelebi, İzmir, Turkey. Technical procedures, manuscript preparation and writing, final approval.; II PhD, Research Assistant, Department of Periodontology, Faculty of Dentistry, University of Bingöl, Turkey. Technical procedures, manuscript preparation and writing, final approval.; III PhD, Assistant Professor, Department of Periodontology, Faculty of Dentistry, University of Dicle, Diyarbakir, Turkey. Technical procedures, manuscript preparation and writing, final approval.; IV PhD, Professor, Department of Anatomy, Faculty of Medicine, University of Dicle, Diyarbakir, Turkey. Technical procedures, histopathological examinations, manuscript preparation and writing, final approval.

**Keywords:** Reishi, Skull, Osteopontin, Osteonectin, Matrix Metalloproteinase 9, Rats

## Abstract

**Purpose:**

*Ganoderma lucidum*, a kind of mushroom used for its antioxidant, anti-inflammatory, and immunomodulatory activities, was investigated in the present study for its possible healing effect on calvarial defects with bone grafts.

**Methods:**

Wistar male rats (*n* = 30) were divided into 3 groups: 1) the control (defect) group (*n* = 10), 2) defect and graft group (*n* = 10), and 3) defect, graft, and *G. lucidum* treated group (*n* = 10). The *G. lucidum* was administered to the rats at 20 mL/kg per day via gastric lavage.

**Results:**

In the defect and graft group, osteonectin positive expression was observed in osteoblast and osteocyte cells at the periphery of the small bone trabeculae within the graft area. In the defect, graft, and *G. lucidum* treated group, osteonectin expression was positive in the osteoblast and osteocyte cells and positive osteonectin expression in new bone trabeculae. The expression of matrix metalloproteinase-9 (MMP-9) was positive in the inflammatory cells, fibroblast cells, and degenerated collagen fibril areas within the defect area.

**Conclusion:**

This study shows that, with its antioxidant and anti-inflammatory properties, *G. Lucidum* is an important factor in the treatment of calvarial bone defects.

## Introduction

Bone defects occur as a result of trauma, ageing, congenital anomalies, neoplasms, and infectious conditions. The treatment of bone defects may be complicated with the effect of tissue disadvantages, and recovery may be delayed. Several procedures have been used to stimulate bone regeneration in osseous defects in the craniofacial area^1^. To enhance bone regeneration in bone defects, researchers have studied different types of therapies, such as autogenous bone grafts, allogeneic banked bone, demineralized matrix pastes, ceramic scaffolds, synthetic materials^2^, medicinal herb treatment (such as *Salvia miltiorrhiza*)^3^, platelet-rich plasma application^4^, and, especially in calvarial bone defects, laser and ozone therapy^5^ ^,^ ^6^ and Danshen and Ge Gan herbal extract treatment^7^.

*Ganoderma lucidum* (aphyllophoromycetideae from the family Polyporaceae) is a mushroom that has been used in traditional Chinese medicine for many years^8^. In addition, *G. lucidum* has been reported as an important source of bioactive compounds, such as polysaccharides, triterpenoids, and proteins, which are used to prevent or treat various human diseases, such as cancer, immunological disorders, neurodegenerative diseases, hepatitis, hypertension, chronic bronchitis, bronchial asthma, and others^9^ ^,^ ^10^.

Previous studies have reported that *G. lucidum* performs antioxidant, hypoglycaemic, anti-inflammatory, anti-tumour, and immunomodulatory activities^11^ ^–^ ^13^. In addition, *G. lucidum* acts to reduce oxidative stress and promote neuroprotective effects^14^, inducing neuronal differentiation^15^ and protecting against cerebral ischaemic injury by inhibiting apoptosis^16^. Additionally, *G. lucidum* extract reduces the expressions of proinflammatory and cytotoxic factors from the activated microglia and effectively protects the dopaminergic neurons against inflammatory and oxidative damage^17^. Zhang et al. suggested that *G. lucidum* preserves the injured spinal motor neuron expression levels of the proteins that play important roles in axonal regeneration^18^. These results imply that the polysaccharide extracts isolated from *G. lucidum* have neural protection and antioxidant properties.

Osteopontin is one of the noncollagenous proteins present in the bone matrix and is involved in a wide range of processes, such as cell adhesion, cell signalling, migration, inflammation^19^, and osteoclast distribution to bone surfaces during bone resorption^20^ ^,^ ^21^. Osteopontin is expressed by bone cells by osteocytes that are exposed to mechanical stress^22^. Osteonectin is expressed by osteoblasts and odontoblasts^23^ and plays a role in the mineralisation of bone and cartilage matrices^24^, cell-matrix interactions, and collagen binding. Osteonectin also increases the production and activity of matrix metalloproteinases; thus, it is used as an indicator of new bone formation^25^.

Matrix metalloproteinase-9 (MMP-9) is a matrix metalloprotein capable of inducing chondrocyte damage through osteoarthritis and collagen degradation and can facilitate angiogenesis in osteoarthritis tissues that induce pannus^26^. The transcription of MMP-9 can be highly induced by numerous agents, including growth factors, cytokines, cell-cell and cell-ECM adhesion molecules, and agents that alter the cell shape^27^.

This study was designed to examine the possible protective effects of *G. lucidum* against calvarial bone defects using immunohistochemical methods. Thus, we aimed to investigate the effects of *G. lucidum* administration on calvarial bone defects with graft material regarding whether it can be used as a regenerative agent in osteoinductive reaction and new bone formation.

## Methods

The investigation was conducted in accordance with the *Guide for the Care and Use of Laboratory Animals* published by the US National Institutes of Health (NIH Publication no. 85-23, revised 1996).

In this study, 30 Wistar male rats weighing 280-300 (gr) were used. The rats were housed individually in suitable cages, at a temperature of 22ºC ± 2ºC and in 12 hours of dark, 12 hours of light. Animals were fed with standard laboratory food and water. All rats were healthy at the end of the analysis, and no distinction in nourishment, water consumption, or body-weight increment among the experimental and control rats was noted. Ganoderma lucidum showed no toxic effect on rats at 20 mL/kg per day via gastric gavage. And, there was no rat death in these experimental groups.

In our study, 3 groups were formed.

1. **Control (defect) group:** Ten rats were treated with an 8 mm calvarial bone defect, and the wound was sutured without any treatment. The rats were sacrificed at the end of the fourth week.
2. **Defect and graft group:** In 10 rats, alloplastic bone grafts were applied to the defect by creating an 8 mm calvarial bone defect. The rats were sacrificed at the end of the fourth week.
3. **Defect, graft, and *G. lucidum* treated group:** Alloplastic bone graft application and *G. lucidum* treatment were performed for 10 rats. The rats were sacrificed at the end of the fourth week.

### Calvarial defect procedure

The animals were anaesthetized intraperitoneally with 3 mg/kg xylazine (Rompun^®^ 2%, Bayer Kimya San. Ltd. Sti., Istanbul, Turkey) and 90 mg/kg ketamine HCl (Ketalar^®^, EWL Eczacibasi Warner Lambert Ilaç Sanayi ve Ticaret A.S., Istanbul, Turkey)^28^. The skin was incised open to the frontal bone. A periosteal flap was removed with a thin elevator. Surgical sites were exposed with an incision through the skin and periosteum at the midline of the calvaria. The periosteal flap was removed with a thin periosteal elevator, and a specially designed trephine bur was created with a circular full-thickness bone defect with a diameter of 0.8 mm on the midline.

### Graft application

The allograft material that was placed in the defect area of Groups 2 and 3 was Biograft^®^ HT (IFGL Bio Ceramics), which contains 40% β-tricalcium phosphate with 60% porous biphasic synthetic hydroxyapatite. This material is an alloplast with a granule size of 350 to 500 μm with osteoconductive properties. The subcutaneous tissue was sealed with 6/0 vicryl sutures, and the skin was allowed to heal.

### Ganoderma lucidum administration

The *G. lucidum* fungus mixture (water-soluble) was provided by Shandong Si Wei Co., Ltd. (Heze, Shandong Province, China; licence No. Z200220083). The preparation of the *G. lucidum* fungus mixture involved the inoculation of a pure culture of *G. lucidum* mycelia into a solid culture medium (composed of bagasse and defatted rice bran), and it was cultured until just before the formation of the fruit body (for 3–4 months). The air-dried *G. lucidum* fruit bodies were extracted with hot water and were sterilized by filtration, as described previously^16^ ^,^ ^29^. In addition, *G. lucidum* was administrated to rats at 20 mL/kg per day via gastric gavage (polysaccharides at 2 mg/mL)^30^.

### Histologic examinations

At the end of the study, the animals were anaesthetized intraperitoneally with 3 mg/kg xylazine and 90 mg/kg ketamine HCl. Then, all animals were sacrificed by decapitation. The skin, as well as all of the soft tissue surrounding the calvarial bone, was removed. The samples were fixed with 10% neutral buffered formalin solution and decalcified with 5% ethylenediaminetetraacetic acid (EDTA). After rinsing with tap water, the samples were dehydrated in increasing concentrations of ethanol and were embedded in paraffin. Tissue sections of 4 to 6 μm thickness (RM2265 rotary microtome; Leica, Germany) were prepared in the transverse plane and were stained using hematoxylin-eosin (H-E) staining for light microscopy examination.

The H-E staining procedure was as follows. After the deparaffinising procedure on the sections with 2 changes of xylene for 10 minutes each, they were rehydrated in 2 changes of absolute alcohol for 5 min each. Then, 95% alcohol was applied for 2 min, and 70% alcohol was applied for 2 min. Next, the sections were washed briefly in distilled water. Then, they were stained in a Harris hematoxylin solution for 8 min. After washing in running tap water for 5 min, the sections were differentiated in 1% acid alcohol for 30s. After bluing in 0.2% ammonia water for 30 s, they were washed in running tap water for 5 min and rinsed in 95% alcohol. They were counterstained in an eosin-phloxine solution for 30 s and dehydrated through 95% alcohol and 2 changes of absolute alcohol for 5 min each. They were cleared in 2 changes of xylene for 5 min each and mounted with a xylene-based mounting medium.

### Immunohistochemical staining

Samples of calvaria bone were fixed with 10% formaldehyde solution, decalcified with 5% EDTA (calvaria bone tissue was decalcified in 15 days), dehydrated in a graded series of ethanol, and then embedded in paraffin wax. Then, 4–5 μm thick sections were cut with a microtome (Leica, Germany) and placed on coated slides. The sections were brought to distilled water and washed three times for 5 min in phosphate-buffered saline (PBS, pH 7.4; catalogue number # 10010023, Thermo Fisher Scientific, US). To unmask antigen sites, the slides were incubated with EDTA solution in a microwave for 110 min 3 times at 90°C. The sections were washed 3 times for 5 min in PBS and incubated with hydrogen peroxide (catalogue #TA-015-HP, Thermo Fisher Scientific, US) for 20 min. Ultra V block (TA-125-UB, Thermo Fisher Scientific, US) was applied to the sections for 8 min prior to the addition of the primary antibodies, which were left on overnight (osteonectin; SPARC monoclonal antibody, catalogue # 33-5500, 1:100; osteopontin monoclonal antibody, catalogue #MA5-17180, 1:100; and MMP-9 monoclonal antibody, catalogue #MA5-13595, 1:200, all from Thermo Fisher Scientific, US). The sections were washed 3 times for 5 min in PBS and then were incubated with biotinylated secondary antibody (catalogue #TP-125-BN, Thermo Fisher Scientific, US) for 14 min. After washing with PBS, streptavidin peroxidase (catalogue #TS-125-HR, Thermo Fisher Scientific, US) was applied to the sections for 15 min. The sections were washed 3 times for 5 min in PBS. Diaminobenzidine (catalogue #TA-012-HDC, Thermo Fisher Scientific, US) was applied to the sections for up to 20 min as a chromogen. The control slides were prepared using the same procedure, without primary antibodies. Counterstaining was done using Harris's haematoxylin for 45s, dehydrated through ascending alcohol and cleared in xylene (Product Number: HHS32 Sigma, hematoxylin solution, Harris modified, Sigma-Aldrich, 3050 Spruce Street, Saint Louis, MO, 63103, USA). The slides were mounted with Entellan^®^ (lot: 107961, Sigma-Aldrich, St. Louis, MO, USA) and examined under a light microscope (Olympus, Germany).

### Semi-quantitative scoring of histopathological parameters

A semi-quantitative scoring was determined by examining osteoblast, osteocyte, and osteoclast cells in the bone tissue. During the obtaining of histological sections after routine histological follow-up, 15 different areas were scanned for each slide, and the mean value of the 10 randomly selected cells was calculated. As a result of these averages, 10 mean scores were obtained for each group of animals, and these data were analysed statistically. Decimals were converted to integers whereas the averages were obtained before statistical analysis (Table 1). Similar semi-quantitative methods have been used in previous histochemical studies of bone tissue^31^ ^–^ ^33^.

**Table 1:** Statistical results of groups using the Kruskal–Wallis and post-hoc Dunnett T3 tests.

Parameters	Groups	n	Mean ± SD	Mean Rank	p-value
***Inflammation***	*(1) Control*	*10*	3.40 ± 0.51	*23.40*	* *(2)* * *(3)*
	*(2) Defect and Graft*	*10*	2.70 ± 0.48	*17.60*	* *(1)* * *(3)*
	*(3) Defect, Graft, and G. Lucidum*	*10*	0.60 ± 0.51	*5.50*	* *(1)* * *(2)*
***Congestion in blood vessels***	*(1) Control*	*10*	3.50 ± 0.52	*23.25*	* *(2)* * *(3)*
	*(2) Defect and Graft*	*10*	2.80 ± 0.63	*17.60*	* *(1)* * *(3)*
	*(3) Defect, Graft, and G. Lucidum*	*10*	0.80 ± 0.63	*5.65*	* *(1)* * *(2)*
***New bone formation***	*(1) Control*	*10*	1.00 ± 0.47	*6.25*	* *(2)* * *(3)*
	*(2) Defect and Graft*	*10*	2.30 ± 0.67	*15.15*	* *(1)* * *(3)*
	*(3) Defect, Graft, and G. Lucidum*	*10*	3.80 ± 0.42	*25.10*	* *(1)* * *(2)*
***OP expression in osteoblasts***	*(1) Control*	*10*	0.50 ± 0.40	*5.75*	* *(2)* * *(3)*
	*(2) Defect and Graft*	*10*	2.40 ± 0.69	*16.25*	* *(1)* * *(3)*
	*(3) Defect, Graft, and G. Lucidum*	*10*	3.60 ± 0.51	*24.50*	* *(1)* * *(2)*
***OP expression in osteocytes***	*(1) Control*	*10*	0.70 ± 0.48	*5.50*	* *(2)* * *(3)*
	*(2) Defect and Graft*	*10*	2.50 ± 0.52	*17.00*	* *(1)* * *(3)*
	*(3) Defect, Graft, and G. Lucidum*	*10*	3.50 ± 0.70	*24.00*	* *(1)* * *(2)*
***OP expression in osteoclasts***	*(1) Control*	*10*	3.50 ± 0.70	*24.55*	* *(2)* * *(3)*
	*(2) Defect and Graft*	*10*	2.00 ± 0.81	*15.55*	* *(1)* * *(3)*
	*(3) Defect, Graft, and G. Lucidum*	*10*	0.60 ± 0.51	*6.40*	* *(1)* * *(2)*
***ON expression in osteoblasts***	*(1) Control*	*10*	0.92 ± 0.34	*6.15*	* *(2)* * *(3)*
	*(2) Defect and Graft*	*10*	2.80 ± 0.72	*17.65*	* *(1)* * *(3)*
	*(3) Defect, Graft, and G. Lucidum*	*10*	3.80 ± 0.84	*25.30*	* *(1)* * *(2)*
***ON expression in osteocytes***	*(1) Control*	*10*	0.82 ± 0.46	*7.10*	* *(2)* * *(3)*
	*(2) Defect and Graft*	*10*	2.94 ± 0.26	*17.45*	* *(1)* * *(3)*
	*(3) Defect, Graft, and G. Lucidum*	*10*	4.10 ± 0.74	*24.90*	* *(1)* * *(2)*
***ON expression in osteoclasts***	*(1) Control*	*10*	3.10 ± 0.92	*26.25*	* *(2)* * *(3)*
	*(2) Defect and Graft*	*10*	1.95 ± 0.85	*15.10*	* *(1)* * *(3)*
	*(3) Defect, Graft, and G. Lucidum*	*10*	1.15 ± 0.44	*5.20*	* *(1)* * *(2)*
***MMP-9 expression***	*(1) Control*	*10*	2.50 ± 0.70	*16.55*	* *(2)*
	*(2) Defect and Graft*	*10*	1.90 ± 0.56	*9.30*	* *(1)* * *(3)*
	*(3) Defect, Graft, and G. Lucidum*	*10*	2.90 ± 0.56	*20.65*	* *(2)*

* *p* < .01; ON: osteonectin, OP: osteopontin OB: osteoblast, OS: osteocyte, OC: osteoclast.

Different superscripts on the *p*-value column show significant differences between groups.

### Statistical analysis

Statistical analyses were performed with SPSS 24.0 for Windows. The data of the parameters were evaluated with the non-parametric Kruskal–Wallis test, and multiple comparisons were evaluated using the post-hoc Dunnett T3 test. The results of the scores were given in Table 1 as the mean rank and mean ± standard deviation (*SD*). The mean ± *SD* values were obtained as a result of the descriptive post-hoc test. The results were considered statistically significant for *p*<.05 (Table 1).

## Results

The histopathological and immunohistochemical results of the present study were evaluated under a light microscope. We compared the histopathological findings in the control and experimental groups (Table 1).

### Histopathologic findings

**Figure 1:**
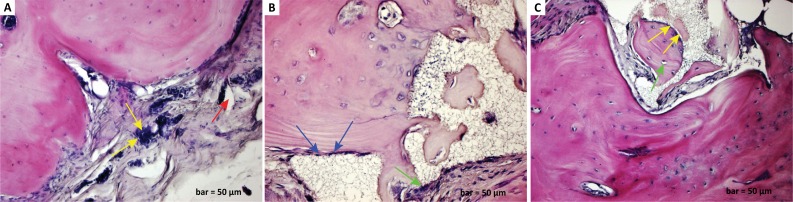
**a.** Haematoxylin-eosin staining (defect group). Inflammatory cell infiltration in the connective tissue (*yellow arrows*), increase in osteoclast cells, congestion with dilatation of blood vessels (*red arrow*), decreased numbers of osteoblast and osteocyte cells, and degeneration of connective tissue fibres. **b.** Haematoxylin-eosin staining (defect and graft group). Mitotic activity in the osteoblast cells at the periphery of the calvarial bone (*blue arrows*) and decrease in inflammatory cells between the defect and graft site with a reduction of osteoclast cells (*green arrow*). **1c.** Haematoxylin-eosin staining (defect, graft, and Ganoderma lucidum treated group). Increase in bone matrix and bone trabeculae and increase in osteoblast cells in the calvarial bone periphery (*yellow arrows*), maturation in osteocyte cells (*green arrow*), and new bone formation in osteon structures within the bone trabeculae. Scale bar=50 μm.

In the control (defect) group, inflammatory cell infiltration in the connective tissue was observed in the form of aggregate-forming cells, while an increase in osteoclast cells was observed. We observed congestion with the dilatation of blood vessels within the defect area. The numbers of osteoblast and osteocyte cells were decreased, and degeneration in the connective tissue fibres was found (Fig. 1a). In the defect and graft group, mitotic activity started in the osteoblast cells at the periphery of the calvaria bone. A decrease in inflammatory cells was observed between the defect and graft site with a reduction of osteoclast cells. It was observed that the osteocytes embedded in lacuna were prominent together with the osteoblast cells located in the periphery of the immature new bone trabeculae within the graft area (Fig. 1b). In the defect, graft, and *G. lucidum* treated group, a significant increase in the bone trabecular structure and matrix was observed. However, there was an increase in osteoblast and osteocyte cells located at the periphery of the calvaria bone. New bone formation was also observed in osteon structures within the bone trabeculae (Fig. 1c).

### Immunohistochemical findings

**Figure 2:**
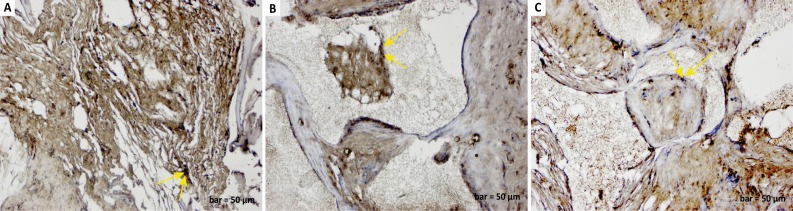
**a.** Osteonectin immunostaining (defect group). Positive osteonectin expression in degenerated collagen fibres, inflammatory cells, and osteoclast cells in the defect area (*yellow arrows*), and negative osteonectin expression in osteoblast cells. **b.** Osteonectin immunostaining (defect and graft group). Positive osteonectin expression in osteoblasts (*yellow arrows*) and osteocytes in the graft area. **c.** Osteonectin immunostaining (defect, graft, and Ganoderma lucidum treated group). Positive osteonectin expression in osteoblasts, osteocytes, and new bone trabeculae (*yellow arrows*), increased in number along with calvarial bone, and constriction of graft areas. Scale bar=50 μm.

In the control (defect) group, osteonectin positive expression was observed in degenerated collagen fibres, inflammatory cells, and osteoclast cells in the defect area, and negative osteonectin expression was seen in osteoblast cells (Fig. 2a). In the defect and graft group, osteonectin positive expression was observed in osteoblast cells and osteocyte cells at the periphery of the small bone trabeculae within the graft area (Fig. 2b). In the defect, graft, and *G. lucidum* treated group, osteonectin expression was positive in osteoblast and osteocyte cells and new bone trabeculae, which increased in number along with calvarial bone with the constriction of the graft areas (Fig. 2c).

**Figure 3:**
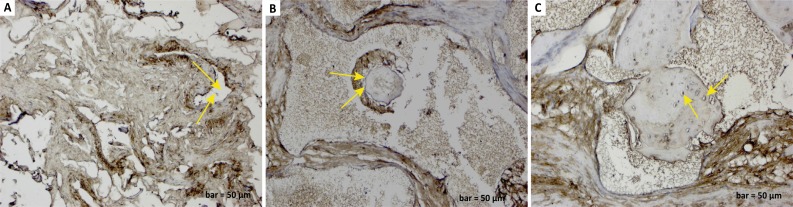
**a.** Osteopontin immunostaining (defect group). An increase in osteopontin expression in osteoclast cells and inflammatory cells around enlarged blood vessels (*yellow arrows*). **b.** Osteopontin immunostaining (defect and graft group). Positive osteopontin expression in osteoblasts and osteocytes at the periphery of calvarial bone (*yellow arrows*) trabeculae and new bone trabeculae within the graft area. **c.** Osteopontin immunostaining (defect, graft, and Ganoderma lucidum treated group). Positive osteopontin expression in osteoblasts, osteocytes (*yellow arrows*), and osteons with osteoblastic activity. Scale bar=50 μm.

There was an increase in osteopontin expression in the osteoclast cells in inflammatory cells around enlarged blood vessels in the defect group (Fig. 3a). In the defect and graft group, osteopontin positive expression was observed in osteoblast cells and some osteocyte cells located at the periphery of calvarial bone trabeculae and at the periphery of new bone trabeculae within the graft area (Fig. 3b). In the defect, graft, and *G. lucidum* treated group, osteopontin expression was observed in osteoblast and osteocyte cells and osteons with osteoblastic activity (Fig. 3c).

**Figure 4:**
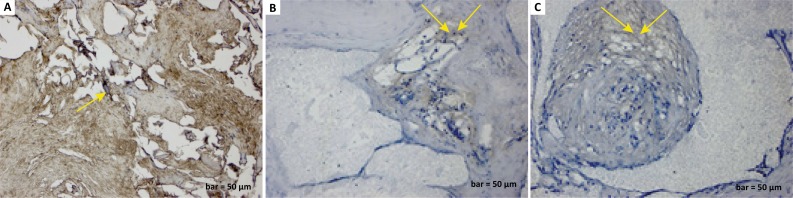
**a.** MMP-9 immunostaining (defect group). Positive MMP-9 expression in inflammatory cells, fibroblast cells, and degenerated collagen fibril areas (*yellow arrow*). **b.** MMP-9 immunostaining (defect and graft group). Positive MMP-9 reaction in fine collagen fibrils around blood vessels (*yellow arrows*). **c.** MMP-9 immunostaining (defect, graft, and Ganoderma lucidum treated group). Positive MMP-9 expression in bone matrix and collagen fibrils (*yellow arrows*) with the maturation of bone trabeculae. Scale bar=50 μm.

In addition, MMP-9 expression was positive in inflammatory cells, fibroblast cells, and degenerated collagen fibrils areas in the defect group (Fig. 4a). A positive MMP-9 reaction was observed in fine collagen fibrils around the blood vessels with some inflammatory cells in the defect and graft group (Fig. 4b). Moreover, MMP-9 expression was positive in the bone matrix and collagen fibrils with the maturation of bone trabeculae in the defect, graft, and *G. lucidum* treated group (Fig. 4c).

## Discussion

Bone regeneration in calvarial defects in experimental studies is an important model in implant application and treatment. Various methods, derivatives, and remedies are being used for regeneration in bone defects^4^ and for calvarial bone defects^7^. It was thought that graft applications could be a suitable model for determining the bone regenerative effects in comparison with other experimental bone defects. Regeneration of bone, infiltration of granulation tissue, and remodelling of osteogenic cells with proliferation and healing occurred herein. In our study, inflammatory cell infiltration, aggregate-forming cells, and osteoclast cells increased in the defect area. Our histopathological findings included dilatation of blood vessels, decreased numbers of osteoblast and osteocyte cells, and degeneration of connective tissue fibres (Fig. 1a). Different bone graft materials have been used for bone regeneration, closure of osteotomy openings, and alveolar augmentation by oral and maxillofacial surgeons^34^. In our study, an alloplastic graft material consisting of a combination of 350 to 500μm diameter porous biphasic hydroxyapatite granules and β-tricalcium phosphate granules was used. In the defect and graft group, there was an increase in mitotic activity in osteoblast cells around the calvarial bone, a decrease in osteoclast cells, and a decrease in inflammatory cells between the defect and graft region. The study found osteoblast cells surrounding the immature new bone trabeculae in the graft area and osteocyte cells embedded in the lacuna (Fig. 1b).

Herbal medicines have been used for centuries to treat a variety of diseases and conditions involving fractured bone healing, bone formation^35^, and other bone diseases, such as osteoporosis^36^. In the past few years, many studies have demonstrated the beneficial effects of *G. lucidum*. Gokce et al. evaluated *G. Lucidum* for antioxidant, anti-inflammatory, and anti-apoptotic activity and neuroprotective effects in a spinal cord injury model in rats and showed that treatment with *G. lucidum* polysaccharides improves the functional and biochemical results^37^.

Özevren et al. demonstrated that *G. lucidum* treatment after brain injury could be an alternative treatment to decrease inflammation and oedema, preventing neuronal and glial cell degeneration with analyses of p38 mitogen-activated protein kinase, vascular endothelial growth factor, and cluster of differentiation 68 expression levels^38^. Another study by Özevren *et al*.^39^ on rats with traumatic brain injury showed that *G. lucidum* polysaccharides may play a certain role for expression of apoptosis-associated proteins and, in this way, have neuroprotective effects. Chen *et al*.^40^ studied the protective roles of polysaccharides from *G. lucidum* on bleomycin-induced pulmonary fibrosis in rats. They indicated that *G. lucidum* inhibited pulmonary fibrosis by reducing inflammatory cell infiltration and collagen deposition in a histopathological manner. Ekinci *et al*.^41^ found that *G. lucidum* reduced spinal cord injury-induced oxidative stress and exerted neuroprotection by inhibiting lipid peroxidation and GSH depletion in spinal cord injuries. According to Ma *et al*.^42^, *G. lucidum* extracts may be used even in the treatment of diabetes because the polysaccharide, proteoglycan, protein, and triterpenoid content of *G. lucidum* has hypoglycaemic effects. In a study by Lacin *et al*.^6^, a calvarial bone defect was created in a rat model, and ozone treatment was applied with a graft. They concluded that the ozone treatment promoted graft consistency and induced angiogenesis, cell proliferation, and matrix formation for osteoblastic activity. In the defect, graft, and *G. lucidum* treated group, an increase in osteoblast cells, maturation of osteocyte cells, increase in the bone matrix with a narrowing of the graft area, maturation of osteon structures in bone trabeculae, and formation of new bone were clearly seen in the sections (Fig. 1c).

Osteonectin is an extracellular matrix glycoprotein with noncollagenous acidic properties and is known to be localized in mineralized bone trabeculae^24^. It is synthesized by osteoblasts at higher levels in the matrix than in the cells of the bone matrix. It is found in periodontal ligaments, semen, bone, fibroblasts, and osteoblast cells. Osteonectin was presented to link the bone mineral and collagen that may initiate active mineralization in normal skeletal tissue^25^. In our study, the bone matrix structure was impaired after the calvarial defect, and as a result of this, a decrease in osteonectin expression was evident in osteoblasts and osteocytes (Fig. 2a). Bone trabeculae began to develop due to the development of the bone matrix with the graft application. Osteoblast cell and osteocyte activity became clear, and osteonectin expression began to show a positive reaction (Fig. 2b). In addition, *G. lucidum* treatment increased osteonectin expression. Treatment with *G. lucidum* seemed to accelerate osteoblast cell development by increasing the bone matrix, and it began to improve osteocyte cell transformation and shape new bone trabeculae (Fig. 2c). In addition, extracellular matrix glycoprotein osteonectin is considered to function in osteocyte transformation by improving osteoblastic activity in the regulation of collagen fibrils at the bone mineralization stage.

Osteopontin has been shown to mediate osteoclast development by mediating cell-cell contact between osteoblastic cells and osteoclast progenitors. Osteopontin has been reported to increase the effect of paracrine cytokines produced by stromal/osteoblastic cells, thus promoting the proliferation or differentiation of haematopoietic precursors. Osteopontin was demonstrated to be functional in cell adhesion, cell signalling and migration, and inflammation^19^. Osteopontin is expressed in osteoblasts and osteocytes in osteoclasts, the cells responsible for bone remodelling^43^. In the defect group, inflammatory cells and osteopontin positive reaction were observed in osteoclast cells (Fig. 3a). The osteopontin reaction was observed in osteoblast cells and some osteocyte cells in the newly developed bone trabeculae in the graft area (Fig. 3b). Osteopontin expression and new bone formation have been observed with the increase of osteoblast activity in the defect, graft, and *G. lucidum* treated group (Fig. 3c).

Moreover, MMP-9 is expressed both by bone marrow-derived myeloid cells and osteoclasts that are involved in the inflammatory response and extracellular matrix remodelling during bone repair^44^ ^,^ ^45^. Colnot *et al*.^44^ showed that MMP-9 operates both during the inflammatory and bone remodelling phases of repair. In another study, MMP-9 has been shown to mediate inflammatory and progenitor cell responses to mechanical stimuli during bone repair. It has been reported that MMP-9 coordinates these events by differentiation of inflammation and skeletal progenitors under different mechanical stimuli^46^.

In this study, MMP-9 expression in collagen fibril and inflammatory cells in the degenerated area showed a positive reaction after the calvarial defect (Fig. 4a). Following the graft application, reduced inflammation around the blood vessels as well as collagen repair and early osteoblastic activity were notable (Fig. 4b). Observations of *G. lucidum* treatment showed that the extracellular matrix was positively stained with MMP-9 in the collagen fibrils of the new bone matrix where the inflammation was decreased (Fig. 4c). Therefore, MMP-9 was thought to act as a mediator of inflammation involved in fracture repair, stimulating the rapid fusion of grafts that are effective on the structure of collagen fibres and inducing extracellular matrix development in the bones.

## Conclusions

In experimental studies, a large number of different materials, techniques, and solutions have been studied for the treatment of calvarial bone defects so far. Despite the results, there are a few limitations. The expression and effect levels of osteopontin, osteonectin, and MMP-9 proteins at the cell level were determined in the graft-induced rats with a calvarial defect model.

## References

[1] Bosch C, Melsen B, Gibbons R, Vargervik K (1996). Human recombinant transforming growth factor-beta 1 in healing of
calvarial bone defects. J Craniofac Surg.

[2] Szpalski C, Barr J, Wetterau M, Saadeh PB, Warren SM (2010). Cranial bone defects: current and future
strategies. Neurosurg Focus.

[3] Chin A, Yang Y, Chai L, Wong RW, Rabie AB (2011). Effects of medicinal herb Salvia miltiorrhiza on osteoblastic
cells in vitro. J Orthop Res.

[4] Peng W, Kim IK, Cho HY, Seo JH, Lee DH, Jang JM, Park SH (2016). The healing effect of platelet-rich plasma on xenograft in
peri-implant bone defects in rabbits. Maxillofac Plast Reconstr Surg.

[5] Kazancioglu HO, Ezirganli S, Aydin MS (2013). Effects of laser and ozone therapies on bone healing in the
calvarial defects. J Craniofac Surg.

[6] Laçin N, Kaya B, Deveci E, Kadiroğlu ET, Aktaş A, Yalçin M, Uysal E (2018). Comparative evaluation of ozone treatment in critical size bone
defects reconstructed with alloplastic bone grafts. Int J Clin Med.

[7] Lee DH, Kim IK, Cho HY, Seo JH, Jang JM, Kim J (2018). Effect of herbal extracts on bone regeneration in a rat calvaria
defect model and screening system. J Korean Assoc Oral Maxillofac Surg.

[8] Bao XF, Wang XS, Dong Q, Fang JN, Li XY (2002). Structural features of immunologically active polysaccharides
from Ganoderma lucidum. Phytochemistry.

[9] Berovic M, Habijanic J, Zore I, Wraber B, Hodzar D, Boh B, Pohleven F (2003). Submerged cultivation of Ganoderma lucidum biomass and
immunostimulatory effects of fungal polysaccharides. J Biotechnol.

[10] Boh B, Berovic M, Zhang J, Zhi-Bin L (2007). Ganoderma lucidum and its pharmaceutically active
compounds. Biotechnol Annu Rev.

[11] Lin ZB, Zhang HN (2004). Anti-tumor and immunoregulatory activities of Ganoderma lucidum
and its possible mechanisms. Acta Pharmacol Sin.

[12] Li F, Zhang Y, Zhong Z (2011). Antihyperglycemic effect of Ganoderma lucidum polysaccharides on
streptozotocin-induced diabetic mice. Int J Mol Sci.

[13] Zhao W, Jiang X, Deng W, Lai Y, Wu M, Zhang Z (2012). Antioxidant activities of Ganoderma lucidum polysaccharides and
their role on DNA damage in mice induced by cobalt-60 gamma-
irradiation. Food Chem Toxicol.

[14] Zhao HB, Wang SZ, He QH, Yuan L, Chen AF, Lin ZB (2005). Ganoderma total sterol (GS) and GS1 protect rat cerebral cortical
neurons from hypoxia/reoxygenation injury. Life Sci.

[15] Cheung WM, Hui WS, Chu PW, Chiu SW, Ip NY (2000). Ganoderma extract activates MAP kinases and induces the neuronal
differentiation of rat pheochromocytoma PC12 cells. FEBS Lett.

[16] Zhou ZY, Tang YP, Xiang J, Wua P, Jin HM, Wang Z, Mori M, Cai DF (2010). Neuroprotective effects of water-soluble Ganoderma lucidum
polysaccharides on cerebral ischemic injury in rats. J Ethnopharmacol.

[17] Zhang R, Xu S, Cai Y, Zhou M, Zuo X, Chan P (2011). Ganoderma lucidum protects dopaminergic neuron degeneration
through inhibition of microglial activation. Evid Based Complement Alternat Med.

[18] Zhang W, Zeng YS, Wang Y, Liu W, Cheng JJ, Chen SJ (2006). Primary study on proteomics about Ganoderma lucidium spores
promoting survival and axon regeneration of injured spinal motor neurons in
rats. Zhong Xi Yi Jie He Xue Bao.

[19] Thurner PJ, Chen CG, Ionova-Martin S, Sun L, Harman A, Porter A, Ager JW, Ritchie RO, Alliston T (2010). Osteopontin deficiency increases bone fragility but preserves
bone mass. Bone.

[20] Reinholt FP, Hultenby K, Oldberg A, Heinegård D (1990). Osteopontin--a possible anchor of osteoclasts to
bone. Proc Natl Acad Sci U S A.

[21] Ikeda T, Nomura S, Yamaguchi A, Suda T, Yoshiki S (1992). In situ hybridization of bone matrix proteins in undecalcified
adult rat bone sections. J Histochem Cytochem.

[22] Bailey S, Karsenty G, Gundberg C, Vashishth D (2017). Osteocalcin and osteopontin influence bone morphology and
mechanical properties. Ann N Y Acad Sci.

[23] Hamann C, Kirschner S, Günther KP, Hofbauer LC (2012). Bone, sweet bone--osteoporotic fractures in diabetes
mellitus. Nat Rev Endocrinol.

[24] Metsäranta M, Young MF, Sandberg M, Termine J, Vuorio E (1989). Localization of osteonectin expression in human skeletal tissues
by in situ hybridization. Calcif Tissue Int.

[25] Termine JD, Kleinman HK, Whitson SW, Conn KM, McGarvey ML, Martin GR (1987). Osteonectin, a bone-specific protein linking mineral to
collagen. Cell Tissue Res.

[26] Wen L, Shin MH, Kang JH, Yim YR, Kim JE, Lee JW, Lee KE, Park DJ, Kim TJ, Park YW, Kweon SS, Lee YH, Yun YW, Lee SS (2016). The relationships between bone mineral density and radiographic
features of hand or knee osteoarthritis in older adults: data from the
Dong-gu Study. Rheumatology (Oxford).

[27] Hu J, Van den Steen PE, Sang QX, Opdenakker G (2007). Matrix metalloproteinase inhibitors as therapy for inflammatory
and vascular diseases. Nat Rev Drug Discov.

[28] Yavas G, Celik E, Yavas C, Elsurer C, Afsar RE (2017). Spironolactone ameliorates the cardiovascular toxicity induced by
concomitant trastuzumab and thoracic radiotherapy. Rep Pract Oncol Radiother.

[29] Gao Y, Zhou S, Wen J, Huang M, Xu A (2002). Mechanism of the antiulcerogenic effect of Ganoderma lucidum
polysaccharides on indomethacin- induced lesions in the rat. Life Sci.

[30] Hu ZL, Wen SG, Yu RJ, Zhu Y (2003). Effects of Ganoderma lucidum fungus mixture on immune enhancement
in mice. Shandong Zhongyiyao Daxue Xuebao.

[31] Miron RJ, Zhang Q, Sculean A, Buser D, Pippenger BE, Dard M, Shirakata Y, Chandad F, Zhang Y (2016). Osteoinductive potential of 4 commonly employed bone
grafts. Clin Oral Investig.

[32] Lee MK, DeConde AS, Lee M, Walthers CM, Sepahdari AR, Elashoff D, Grogan T, Bezouglaia O, Tetradis S, St John M, Aghaloo T (2015). Biomimetic scaffolds facilitate healing of critical-sized
segmental mandibular defects. Am J Otolaryngol.

[33] Erdmann N, Bondarenko A, Hewicker-Trautwein M, Angrisani N, Reifenrath J, Lucas A, Meyer-Lindenberg A (2010). Evaluation of the soft tissue biocompatibility of MgCa0.8 and
surgical steel 316L in vivo: a comparative study in rabbits. Biomed Eng Online.

[34] Beirne OR (1986). Comparison of complications after bone removal from lateral and
medial plates of the anterior ilium for mandibular
augmentation. Int J Oral Maxillofac Surg.

[35] Singh V (2017). Medicinal plants and bone healing. Natl J Maxillofac Surg.

[36] Wang ZQ, Li JL, Sun YL, Yao M, Gao J, Yang Z, Shi Q, Cui XJ, Wang YJ (2013). Chinese herbal medicine for osteoporosis: a systematic review of
random-ized controlled trails. Evid Based Complement Alternat Med.

[37] Gokce EC, Kahveci R, Atanur OM, Gürer B, Aksoy N, Gokce A, Sargon MF, Cemil B, Erdogan B, Kahveci O (2015). Neuroprotective effects of Ganoderma lucidum polysaccharides
against traumatic spinal cord injury in rats. Injury.

[38] Özevren H, İrtegün S, Deveci E, Aşır F, Pektanç G, Deveci Ş (2017). Ganoderma lucidum protects rat brain tissue against trauma-
induced oxidative stress. Korean J Neurotrauma.

[39] Özevren H, Irtegun S, Ekingen A, Tuncer MC, Özkorkmaz EG, Deveci E, Deveci S (2018). Immunoexpression of vascular endothelial growth factor β- cell
ymphoma 2 and cluster of differentiation 68 in cerebellar tissue of rats
treated with ganoderma lucidum. Int J Morphol.

[40] Chen J, Shi Y, He L, Hao H, Wang B, Zheng Y, Hu C (2016). Protective roles of polysaccharides from Ganoderma lucidum on
bleomycin-induced pulmonary fibrosis in rats. Int J Biol Macromol.

[41] Ekinci A, Özevren H, Emre BB, Ekinci C, Deveci S, Deveci E (2018). Neuroprotective effects of ganoderma lucidum on spinal cord
injury. Int J Morphol.

[42] Ma HT, Hsieh JF, Chen ST (2015). Anti-diabetic effects of ganoderma lucidum. Phytochemistry.

[43] Yamate T, Mocharla H, Taguchi Y, Igietseme JU, Manolagas SC, Abe E (1997). Osteopontin expression by osteoclast and osteoblast progenitors
in the murine bone marrow: demonstration of its requirement for
osteoclastogenesis and its increase after ovariectomy. Endocrinology.

[44] Colnot C, Thompson Z, Miclau T, Werb Z, Helms JA (2003). Altered fracture repair in the absence of MMP-9. Development.

[45] Ortega N, Wang K, Ferrara N, Werb Z, Vu TH (2010). Complementary interplay between matrix metalloproteinase-9,
vascular endothelial growth factor and osteoclast function drives
endochondral bone formation. Dis Model Mech.

[46] Wang X, Yu YY, Lieu S, Yang F, Lang J, Lu C, Werb Z, Hu D, Miclau T, Marcucio R, Colnot C (2013). MMP-9 regulates the cellular response to inflammation after
skeletal injury. Bone.

